# BACE1 Inhibition Utilizing Organic Compounds Holds Promise as a Potential Treatment for Alzheimer's and Parkinson's Diseases

**DOI:** 10.1155/2024/6654606

**Published:** 2024-02-22

**Authors:** Razieh Amini, Shadi Moradi, Rezvan Najafi, Mehrdokht Mazdeh, Amir Taherkhani

**Affiliations:** ^1^Research Center for Molecular Medicine, Hamadan University of Medical Sciences, Hamadan, Iran; ^2^Department of Medical Immunology, School of Medicine, Hamadan University of Medical Science, Hamadan, Iran; ^3^Hearing Disorders Research Cerner, Hamadan University of Medical Sciences, Hamadan, Iran

## Abstract

**Background:**

Neurological disorders like Alzheimer's disease (AD) and Parkinson's disease (PD) manifest through gradually deteriorating cognitive functions. An encouraging strategy for addressing these disorders involves the inhibition of precursor-cleaving enzyme 1 (BACE1).

**Objectives:**

In the current research, a virtual screening technique was employed to identify potential BACE1 inhibitors among selected herbal isolates.

**Methods:**

This study evaluated 79 flavonoids, anthraquinones (AQs), and cinnamic acid derivatives for their potential blood–brain barrier (BBB) permeability. Using the AutoDock 4.0 tool, molecular docking analysis was conducted to determine the binding affinity of BBB permeable compounds to the BACE1 active site. Molecular dynamics (MD) simulations were performed to assess the stability of the docked poses of the most potent inhibitors. The interactions between the most effective plant-based inhibitors and the residues within the BACE1 catalytic site were examined before and after MD simulations.

**Results:**

Ponciretin, danthron, chrysophanol, and N-p-coumaroyltyramine were among the highest-ranking BACE1 inhibitors, with inhibition constant values calculated in the nanomolar range. Furthermore, during 10 ns simulations, the docked poses of these ligands were observed to be stable.

**Conclusion:**

The findings propose that ponciretin, danthron, chrysophanol, and N-p-coumaroyltyramine might serve as potential choices for the treatment of AD and PD, laying the groundwork for the creation of innovative BACE1 inhibitors.

## 1. Introduction

Understanding the fundamental mechanisms of aging and their involvement in initiating and advancing neurodegenerative diseases are crucial to developing effective interventions [1]. With age, Alzheimer's disease (AD) has significantly increased, and Parkinson's disease (PD) is another neurodegenerative disorder linked to aging. AD and PD are characterized by the gradual loss of neurons and synaptic connections, resulting in progressive functional decline over time. Despite their distinctive pathology, they are specific neurodegenerative conditions that typically occur sporadically and later in life compared to inherited forms [2]. Neurodegenerative diseases have impacted 50 million people worldwide; no current disease-modifying treatment is available. AD is characterized by the accumulation of tau neurofibrillary tangles and amyloid-*β* (A*β*) extracellular plaques. The preclinical phase of AD is prolonged, with tau lesions and associated neuronal loss first appearing in the subcortical nuclei, followed by cognitive decline and neuropsychiatric symptoms in the limbic regions as the disease progresses. Unlike tau lesions, there is no significant correlation between the distribution of A*β* and symptoms. Apart from age, risk factors for neurodegenerative diseases include cerebrovascular diseases, diabetes, hypertension, obesity, dyslipidemia, and genetic mutations such as triggering receptors expressed on myeloid cells 2 and ApolipoproteinE-*ε*4. Early onset AD, which develops before age 65, accounts for ∼5% of cases. Familial AD is responsible for around 20% of early onset cases, and mutations in presenilin proteins1 (PSEN-1), presenilin proteins2 (PSEN-2), or Amyloid-beta precursor protein (APP) are responsible for less than 1% of all AD cases [3].

*β*-site amyloid precursor protein cleaving enzyme 1 (BACE1) is an aspartyl protease belonging to the pepsin family. Unlike other peptidases in the same family, such as Cathepsin D and E, BACE1 is unique in that it has a transmembrane domain. Although BACE1 is commonly found in specific types of neuronal cells, it is widely expressed throughout the brain, with a significant presence in neurons, oligodendrocytes, and astrocytes. In addition to A*β* plaques surrounding synaptic endings, BACE1 was detected in endosomal vesicles and plasma membranes of healthy synaptic endings [4]. The sequential cleavage of amyloid precursor protein (APP) by BACE1 and *γ*-secretase results in the production and release of the A*β* peptide in the brain. Consequently, amyloidogenic secretases are being studied as potential therapeutic targets for treating AD. Research suggests BACE1 inhibitors may effectively prevent AD progression by reducing A*β* brain accumulation [5]. Moreover, Lange et al. [6] elucidated a notable association between the genetic variant rs638405 within BACE1 and elevated susceptibility to PD. This groundbreaking discovery establishes a fresh and intricate genetic connection between A*β* pathology and the onset of PD.

Research shows distinct natural substances with significant physiological and neuroprotective activity have been found in several marine macroalgae [7]. Several plant-derived secondary metabolites can target the Nrf2/Keap1/ARE pathway and related mediators in AD, including phenolic compounds, alkaloids, terpenoids, carotenoids, and sulfur compounds [8]. The intricate biochemical effects of natural phenolic compounds render them a feasible alternative and supplementary treatment for age-related degenerative diseases. Phenolic compounds increase neurogenesis by stimulating neurotrophic factors like brain-derived neurotrophic factor (BDNF) and activating various pathways, including cAMP response element-binding protein (CREB), calcium/calmodulin-dependent protein kinase II (CAMKII), Na–K–Cl cotransporter, and protein kinases. Additionally, they play a vital role in regulating cell differentiation, survival, growth, and maintenance [9]. Studies on animal models of neurodegenerative diseases have shown that phenolic substances can reduce the loss of dopaminergic and cholinergic synapses [10]. Additionally, donating electrons to the environment allows phenolic compounds to neutralize radicals. Plant phenolics can also inhibit radical reactions by releasing hydrogen radicals into the medium instead of electrons. More than 4,000 flavonoid compounds have been identified from natural sources, making the flavonoid group an essential element of the phenolic chemical family [11].

The ability of flavonoids to electron conjugate and have a diverse range of substituent sites allows them to act as chelating agents and radical scavengers. These polyphenolic compounds are mainly present in the aerial parts of plants [11]. Multiple studies have characterized polyphenols as agents with antioxidant and anticancer properties [12, 13]. Anthraquinone (AQ) metabolites can be found in various plant species, with a higher concentration in families such as *Rubiaceae, Polygonaceae*, and *Rhamnaceae*. AD patients may benefit from AQ-based compounds because they inhibit cholinesterase, reduce protein aggregate formation, and suppress ROS production, which can mitigate the loss of cholinergic function [14]. The BACE1 inhibitory capacity of alaternin was found to be promising by Jung et al. [15] following their investigation of AQs isolated from *Cassia obtusifolia*. Cinnamic acids are bioactive compounds abundant in a diverse range of fruits, vegetables, and whole grains. Their pharmacological functions have been extensively studied, highlighting their anti-inflammatory, antioxidant, antidiabetic, and anticancer effects. They also exhibit various pharmacological properties that can assist in treating different neurological conditions [16, 17].

Legislation enacted in late December 2022, and endorsed by President Joe Biden, has now eliminated the mandatory requirement for animal testing in the evaluation process for new pharmaceuticals seeking approval from the U.S. Food and Drug Administration (FDA). This transformative adjustment, a longstanding aspiration of animal welfare advocacy groups, has the potential to instigate a substantial departure from the longstanding reliance on animal experimentation that has characterized drug development for over eight decades. This momentous shift underscores the need for scientists to embrace alternative methodologies, such as computer modeling, organ chips, and other innovative nonanimal approaches that have emerged and matured over the past 10–15 years [18]. These points to the growing confidence in the reliability and viability of computational results in the realm of drug design and discovery.

An integrated bioinformatics analysis was performed in this study to investigate the neuroprotective effects of flavonoids, AQs, and cinnamic acid derivatives as BACE1 inhibitors. The study involved predicting the BBB permeability of selected compounds, assessing their binding affinity to the BACE1 active site using AutoDock 4.0, and conducting molecular dynamics (MD) simulation to evaluate the stability of docked poses of components with significant binding affinities.

## 2. Materials and Methods

### 2.1. Blood–Brain Barrier (BBB) Permeability

Using the online tool SwisADME (http://www.swissadme.ch/) [19], a total of 79 herbal isolates, comprising 46 flavonoids [20], 21 AQs (18), and 12 cinnamic acid derivatives [21], were assessed for BBB permeability. After identifying the BBB permeable compounds, they were subjected to molecular docking analysis with the BACE1 catalytic site.

### 2.2. BACE1 and Small Molecules Structure Preparation

The Structural Bioinformatics Protein Data Bank (PDB ID: 2ZHV [22]), accessible at https://www.rcsb.org [23], was used to obtain the three-dimensional structure of BACE1 with an x-ray resolution of 1.85 Å. The analysis of the 2ZHV file demonstrated that the protein consisted of one polypeptide chain (chain A) comprising 411 residues. To minimize the protein's energy, the Swiss-pdbViewer version 4.1.0 was used, available at https://spdbv.unil.ch/ [24]. Hassan et al.'s [25] study identified the key residues within the BACE1 active site. Thirteen amino acids, namely Leu30, ASP32, Tyr71, Thr72, Gln73, Gly74, Lys107, Phe108, Ile110, Trp115, Ile118, Asp228, and Gly230, were detected within the BACE1 catalytic site. The BBB permeable ligands were screened to identify potential BACE1 inhibitors and the ligands were subjected to the structural preparation and energy minimization according to previous reports [20, 21, 26]. Eight positive control inhibitors for BACE1 were identified from the DrugBank online database [27], accessed at https://go.drugbank.com. These components were assigned the following DrugBank IDs: DB07206, DB07734, DB07345, DB07303, DB07874, DB07573, DB07110, and DB07346.

### 2.3. Molecular Docking and MD Simulations

A Windows-based computer with an Intel Core i7 CPU, a 64-bit operating system, and 32 GB of RAM was utilized for molecular docking analyses. A more robust computer configuration was used for the MD simulations, featuring a 64 GB DDR5 installed RAM and an Intel 24-Core i9-13900KF processor. Molecular docking was conducted using AutoDock version 4.0, found at http://autodock.scripps.edu. MD simulations were performed using Discovery Studio Client version 16.1.0.15350. The AutoDock utilizes the Lamarckian genetic algorithm to identify the chemical inside the protonated protein. Scientists commonly employ the Gibbs free energy of binding (*G*_binding_) formula to calculate the energy required to maintain a ligand's binding [28, 29]:

(1) $$\begin{array}{c} \Delta G_{\text{binding}}=\text{Intermolecular Energy}+\text{Total Internal Energy}+\text{Torsional Free Energy}-\text{Unbound System's Energy} \end{array}$$

The PDBQT files of the receptor and ligands were also prepared before the docking process [30]. With a spacing of 0.375 Å, the grid box's dimensions were set to *X*-dimension of 60, *Y*-dimension of 56, and *Z*-dimension of 60, while the *X*-center, *Y*-center, and *Z*-center were 72.077, 49.722, and 11.654, respectively.

Polar hydrogens were introduced to the protein, followed by the incorporation of −0.496 Kolman charges to the receptor [31]. The genetic algorithm parameters were set, with a gene mutation rate of 0.02, a crossover rate of 0.8, and a Cauchy distribution variance for gene mutation established at 1.0. Additionally, the criterion for selecting the least fit individual across generations was determined as 10.

Each ligand underwent 100 docking runs, and the most significant cluster's docked model with the lowest *G*-binding energy was selected, utilizing a root-mean-square deviation (RMSD) tolerance of 2.0 A. The top-ranked BACE1 inhibitors were identified based on the inhibition constant values in the nanomolar range and chosen for further analysis, including the assessment of interaction patterns and MD simulations lasting for 100 ns.

In the setup of the MD simulations, the designated parameters were outlined as follows: employing an orthorhombic cell shape, maintaining a minimum distance of 10 Å from the boundaries, utilizing water as the solvent, setting a target temperature of 310 K, adopting the CHARMm force field, implementing the explicit periodic boundary for solvation model, and incorporating a point charge distribution.

During the simulation, the BACE1 complexes with top-ranked inhibitors and a standard drug were examined, and the RMSD of the backbone atoms and root-mean-square fluctuation (RMSF) were calculated. Furthermore, computations were conducted for the receptor's radius of gyration (ROG) and total energy, contributing to heightened result reliability. The BIOVIA Discovery Studio Visualizer version 19.1.0.18287 was employed to present and evaluate the ligands and BACE1 interactions.

## 3. Results

### 3.1. BBB Permeable Compounds

The BBB was crossed by a total of eight flavonoids (ponciretin, chrysin, hemileiocarpin, isoliquiritigenin, glabridin, licochalcone A, flavone, and formononetin), four AQs (danthron, chrysophanol, alizarin, and rubiadin), and six cinnamic acid derivatives (N-p-coumaroyltyramine, caffeic acid phenethyl ester, o-coumaric acid, ferulic acid, p-coumaric acid, and cinnamic acid). Table S1 contains the names of the plant-based compounds assessed for their potential to cross the BBB in this study.

### 3.2. Binding Affinity between BACE1 and Studied Compounds

**Figure 1:**
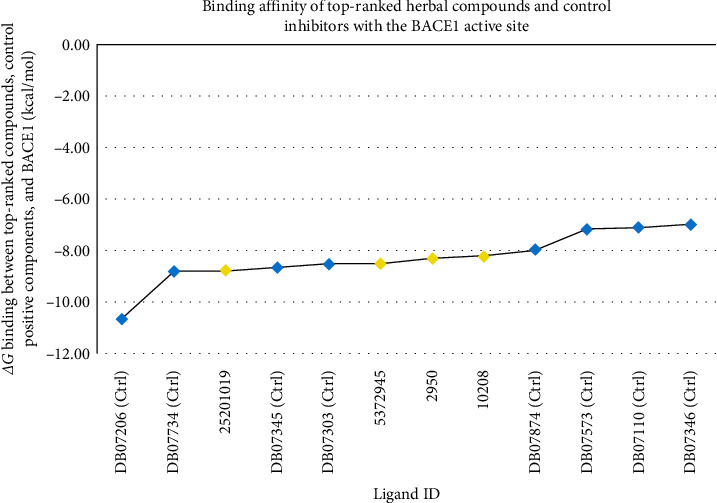
By plotting the compounds' ID on the *x*-axis and their estimated binding energies (kcal/mol) on the *Y*-axis, the study presented the *ΔG*_binding_ values between the most potent herbal inhibitors, positive control compounds, and the BACE1 active site. The blue diamonds represented control compounds, while the yellow spots depicted the top-ranked inhibitors in the study. BACE1, *β*-site amyloid precursor protein cleaving enzyme 1.

The AutoDock 4.0 tool was utilized to gauge the binding affinity of the permeable flavonoids, AQs, and cinnamic acid derivatives to the BACE1 active site. The study's results revealed that four compounds, consisting of one flavonoid, two AQs, and one cinnamic acid derivative, effectively bound to the BACE1 catalytic site at the nanomolar level and were consequently identified as the top-ranked BACE1 inhibitors in this investigation. By computing the *ΔG*_binding_ value, the binding energy between ponciretin, danthron, chrysophanol, N-p-coumaroyltyramine, and the BACE1 active site, were found to be −8.78, −8.30, −8.21, and −8.51 kcal/mol, respectively. Four control inhibitors, DB07206 (6-[2-(1H-INDOL-6-YL) ETHYL] PYRIDIN-2-AMINE), DB07734 (N-(1-benzylpiperidin-4-yl)-4-sulfanylbutanamide), DB07345 (4-(2-aminoethyl)-2-cyclohexylphenol), and DB07303 (N ∼ 3∼–[3-(5-METHOXYPYRIDIN-3-YL) BENZYL] PYRIDINE-2,3-DIAMINE), were found to have inhibition constant values (*K*i) in the nanomolar range. The study's findings are presented in three different formats.  Table 1 illustrates the *Ki* and *ΔG*_binding_ values for 18 herbal isolates and eight positive controls, while Table 2 outlines the energy details between the BACE1 active site and the top-ranked components. In last, Figure 1 compares the binding affinity of the top-ranked compounds and control inhibitors to the BACE1 active site.

### 3.3. Interaction Mode Analysis

The study examined the possible interactions between the top-ranked inhibitors and the BACE1 catalytic site before and after 100 ns MD simulations. The identified interactions included hydrogen bonds, hydrophobic interactions, and electrostatic interactions. During MD simulations, ponciretin and N-p-coumaroyltyramine exhibited the most stable interactions with the residues inside the BACE1 active site.

Before the commencement of MD simulations, ponciretin established a presence involving two hydrogen bonds and four hydrophobic interactions with residues within the BACE1 active site. Upon completing a 100 ns computer simulation, this botanical compound showcased an altered interaction profile, now engaging in three hydrogen bonds and two hydrophobic interactions with residues within the BACE1 catalytic cleft. Notably, one of the hydrogen bonds and two of the hydrophobic interactions exhibited stability throughout the entire MD simulation.

Before initiating MD simulations, N-p-coumaroyltyramine established a complex network comprising four hydrogen bonds, two hydrophobic interactions, and two electrostatic interactions (salt-bridges) with amino acids within the BACE1 active site. Additionally, this compound showcased a refined interaction pattern, engaging in three hydrogen bonds, one hydrophobic interaction, and two electrostatic interactions with residues situated within the BACE1 catalytic cleft. Impressively, one of the hydrogen bonds and two of the electrostatic interactions maintained their stability throughout the entire 100 ns computer simulation. According to previous research, the salt-bridge interaction is widely recognized as one of the most stabilizing interactions between the ligands and receptors [32].

The outcomes were juxtaposed with those obtained from the standard drug, 6-[2-(1H-INDOL-6-YL) ETHYL] PYRIDIN-2-AMINE. The reference drug consistently formed two enduring hydrogen bonds with the residues within the BACE1 catalytic cleft before and following a 100 ns MD simulation.

**Figure 2:**
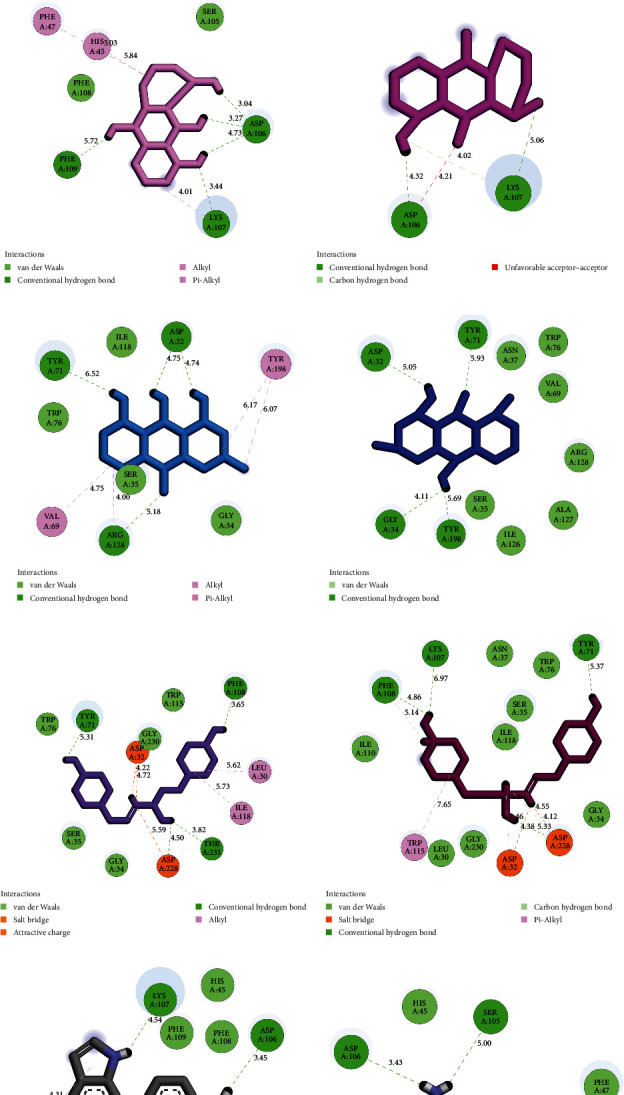
Interaction types among residues within the BACE1 active site and (a) ponciretin, (b) danthron, (c) chrysophanol, (d) N-p-coumaroyltyramine, and (e) DB07206. Left and right images present interactions before and after MD simulations, respectively. BACE1, *β*-site amyloid precursor protein cleaving enzyme 1; DB07206, 6-[2-(1H-INDOL-6-YL)ETHYL]PYRIDIN-2-AMINE; MD, molecular dynamics.

Figure 2 offers two-dimensional representations of the highest-ranked compounds and the reference drug within the BACE1 active site, both pre and post-MD simulations. Conversely, Table 3 elucidates the specific interactions between the top-ranked herbal isolates, the standard drug, and the residues within the BACE1 active site.

### 3.4. MD Simulations

**Figure 3:**
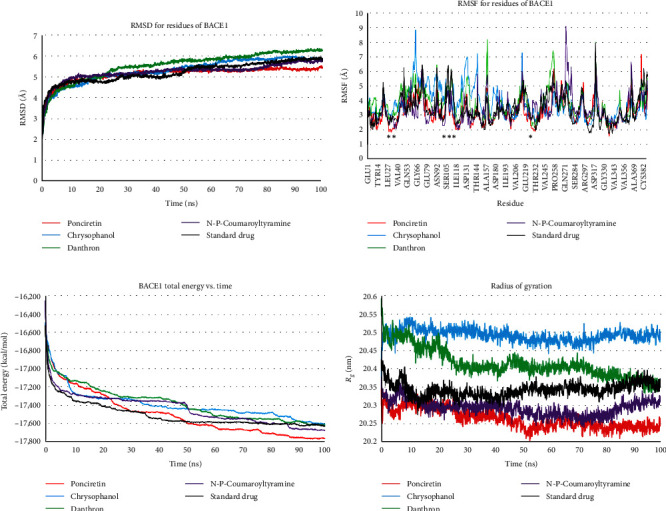
(a) RMSD, (b) RMSF, (c) total energy, and (d) radius of gyration plots of BACE1 backbone atoms in the presence of top-ranked anthraquinones and DB07206 during a 100 ns MD simulation. The *x*-axis presents the residue in the plot (b) and the simulation time in other plots. The *y*-axis presents RMSD, RMSF, total energy, and radius of gyration in plots (a–d), respectively. The location of the asterisks in part (b) is within the protein's active site. RMSD, root-mean-square deviations; RMSF, root-mean-square fluctuation; BACE1, *β*-site amyloid precursor protein cleaving enzyme 1; MD, molecular dynamics; DB07206, 6-[2-(1H-INDOL-6-YL)ETHYL]PYRIDIN-2-AMINE.

Based on the RMSD plot depicted in Figure 3(a), ponciretin emerged as the most robust BACE1 inhibitor, trailed by N-p-coumaroyltyramine and the standard drug. The stability of BACE1 backbone atoms was also observed to persist after approximately 50 ns of simulation, particularly when the BACE1 active site was obstructed by ponciretin. As illustrated in Figure 3(b), the BACE1 active site exhibited diminished fluctuations when interacting with ponciretin and N-p-coumaroyltyramine compared to its interaction with the reference drug.

**Figure 4:**
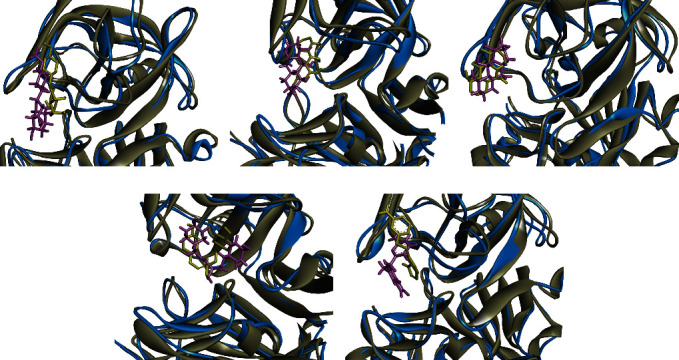
The structures of BACE1 in the presence of (a) ponciretin, (b) chrysophanol, (c) danthron, (d) N-p-coumaroyltyramine, and (e) DB07206 were superimposed after a 100 ns MD simulation. The protein chains before and after the MD analysis are depicted in gray and blue, respectively. The anthraquinones are displayed in yellow and pink before and after the MD simulations. BACE1, *β*-site amyloid precursor protein cleaving enzyme 1; MD, molecular dynamics.

After 100 ns of MD simulations, both BACE1-ponciretin and BACE1-N-p-coumaroyltyramine displayed lower total energy values than the MAPK3-standard drug (Figure 3(c)). Complementing the MD analyses, it was observed that the ROG value for the BACE1 complexes with ponciretin and N-p-coumaroyltyramine was also lower compared to that of the BACE1-standard drug (as illustrated in Figure 3(d)). For a visual representation, the superimposed structures of the BACE1 complexes with the highest-ranked inhibitors and the standard drug were shown both before and after 10 ns of MD simulations in Figure 4.

## 4. Discussion

PD and AD stand as the predominant neurodegenerative conditions related to aging. PD primarily manifests as a movement irregularity, with its distinctive motor traits predominantly arising from the decline of dopaminergic neurons within the substantia nigra pars compacta [33]. Nevertheless, individuals afflicted with PD might encounter an array of nonmotor indications, encompassing certain attributes often linked with AD, such as minor cognitive impairment that frequently evolves into dementia [34]. Past accounts have underscored the noteworthy involvement of BACE1 in both AD and PD.

Computer-aided drug design and virtual screening have become crucial in discovering new lead compounds. By narrowing down the biological target, this approach significantly reduces the time and cost of trials [35]. Recent research has shown that virtual screening techniques centered on plant-derived compounds effectively identify possible inhibitors of biological targets [35]. Due to their advantageous chemical and structural properties, flavonoids have been proposed as inhibitors of BACE1. Nevertheless, no flavonoids have been developed into BACE1 inhibitors that have progressed to the clinical trials [36]. As per recent results, the BACE1 active site showed significant binding affinity to ponciretin, danthron, chrysophanol, and N-p-coumaroyltyramine.

Recent research indicates that poncirin is transformed into ponciretin, the active ingredient of *Poncirus trifoliata*, by gut bacteria both *in vitro* and *in vivo* [37]. The study revealed that ponciretin had the highest binding affinity of all the BBB-permeable flavonoids listed, with a binding energy of −8.78 kcal/mol. In a study by Yusof et al. [36] it was found that flavanones with sugar moieties had a more potent inhibitory effect against BACE1 than those without sugar moieties. The number and position of the glycosidic linkages also influenced the compounds' inhibitory effect. Kang and Kim [38] suggested that poncirin is converted into ponciretin in the gut following ingestion. Both compounds were observed to reduce colitis by inhibiting NF-*κ*B activation via decreased lipopolysaccharide (LPS) binding to macrophages and restoring Th17/Treg cell interactions. The researchers proposed that ponciretin significantly impacted the central nervous system. Recent reports have indicated that all synthesized flavone derivatives had an excellent binding affinity for the BACE1 binding pocket. Their inhibition of BACE1 might be due to interactions with catalytic site residues, such as Asp32 and Asp228 [39]. It has also been reported that *Anacardiaceae* flavonoids obtained from *Rhus verniciflua Stokes* exhibit neuroprotective effects against LPS-activated BV2 microglia and glutamate-induced HT22 hippocampal damage. Various flavonoids have been found to possess therapeutic efficacy against neurodegenerative disorders characterized by oxidative stress and pathological inflammation [40]. Other research has demonstrated that flavonoid extracts can impact various crucial neuroinflammatory markers. *Rosa laevigata Michx*-derived flavonoid-rich extract (FRE) showed neuroprotective effects in rats subjected to cerebral ischemia–reperfusion (I/R). Additionally, the extract exhibited anti-inflammatory effects and reduced oxidative stress and neuronal death. Administration of FRE at doses ranging from 50 to 200 mg/kg resulted in decreased levels of proinflammatory markers (NF-*κ*B, iNOS, COX-2, MMP-9, TNF-*α*, IL-4, IL-6, and IL-1*β*) as well as components of the p-JNK, p-ERK, and p-p38 mitogen-activated protein kinase (MAPK) pathways [41]. In another study, a flavonoid-rich isolate from *Capparis spinosa*, high in rutin and quercetin, was administered to Wistar rats with A*β*-induced Alzheimer's disease for 6 weeks. The treatment decreased the expression of inflammation-related proteins BACE1, APP, PSEN-1, and PSEN-2 in the rats' hippocampi [42]. *In vitro* experiments conducted by Shimmyo et al. [43] revealed that myricetin, kaempferol, morin, quercetin, and apigenin, among other flavonoids, inhibited BACE1 activity and reduced A*β* release from primary cortical neurons. In terms of BACE1 inhibitors, myricetin had the highest IC50 (2.8 *μ*M), followed by quercetin (5.4 *μ*M), kaempferol (14.7 *μ*M), morin (21.7 *μ*M), and apigenin (38.5 *μ*M). Docking studies indicated that flavonoids bind to catalytic sites such as Asp32, Trp198, and Gln73, as well as to the C3–OH group of the C ring.

The results of this work indicate that danthron and chrysophanol had the most significant binding affinity to the BACE1 catalytic site compared to all other AQs tested. Chiou et al. [44] conducted a study suggesting that danthron kills C6 rat glioma cells through the formation of reactive oxygen species, the collapse of mitochondrial transmembrane potential, and the release of cytochrome c, AIF, and Endo G. The study also showed that the apoptotic pathways driven by ROS and mitochondria were effective with danthron. It has been demonstrated that the administration of danthron can inhibit membrane lipid peroxidation and reduce glutathione levels, which can prevent the neurotoxicity caused by *β*-amyloid protein [45]. In a study involving rats with AD-like symptoms, it was found that emodin, an AQ, lowered plaque A*β* levels and tau hyperphosphorylation by reducing BACE1 levels in the hippocampi and enhancing the activity of protein phosphatase 2A (PP2A). Additionally, emodin was found to promote hippocampal neurogenesis and increase synapse-related proteins, reduce oxidative stress through modulation of malondialdehyde/superoxide dismutase (MDA/SOD), and decrease levels of DNA methyltransferases 1/3, among other effects. In AD-like rats, emodin was observed to reduce microglial activation and improve cognitive function and cerebral microvascular integrity by decreasing levels of 5-lipoxygenase (5-LO), IL-6, and TNF-*α* [46]. Another study investigated the anti-AD potential of 19 substances, which included AQs, naphtopyrones, and naphthalene glycosides, by assessing their inhibitory activity against acetylcholinesterase (AChE), butyrylcholinesterase (BChE), and BACE1. The results showed that all substances strongly inhibited AChE, BChE, and BACE1, with cassiaside, alaternin, and emodin being the most potent inhibitors [15]. A study by Li et al. [47] suggested that chrysophanol could improve memory performance in rats developed with A*β*25-35 or D-galactose by suppressing tau hyperphosphorylation.

Among the cinnamic acid derivatives, N-p-coumaroyltyramine exhibited the highest affinity to BACE1 active site, with a binding energy of −8.51 kcal/mol. An experimental study explored the inhibition of AChE and BChE by a new group of cinnamic acid derivatives containing a 1-benzyl-1,2,3-triazole group. The findings revealed that the compound 7b-4 ((E)-3-(3,4-dimethoxyphenyl)-N-((1-(4-fluorobenzyl)-1H-1,2,3-triazole-4-yl)methyl) acrylamide) acrylamide had neuroprotective properties and was influential in inhibiting BACE1 activity [48]. Takahashi and Miyazawa [49] discovered a group of BACE1 inhibitors based on serotonin cinnamamide compounds, published in 2011. N-cinnamoyl serotonin was identified as the most potent molecule, with an IC50 of 86.7 M. The researchers concluded that the functional groups solely influenced the inhibition of BACE1 in the cinnamic acid scaffold [49]. Takao et al. [50] synthesized a group of cinnamic amides and esters of caffeic acid, ferulic acid, and p-coumaric acid in 2017. They investigated their antioxidant activity, acetylcholinesterase, BChE, and monoamine oxidases (MAO) A and B inhibition. The results indicated that the amides had superior DPPH free radical scavenging activity, while the esters were more effective in inhibiting BChE and MAO-B [50]. Researchers synthesized a new class of cinnamic acid compounds containing an N-benzyl pyridinium moiety and examined their multifunctional cholinesterase inhibitory properties against AD. Compound 5l was found to have neuroprotective effects against A*β* (1–42) toxicity in PC12 (rat pheochromocytoma) cells and was able to pass the BBB both *in vitro* and *in vivo* using the BBB specific parallel artificial membrane permeability (PAMPA-BBB) test [51].

Through studying the interplays among premier herbal isolates and residues located within the BACE1 catalytic site, both preceding and after MD simulation, the subsequent aspects could be highlighted:

1. The hydroxyl groups in rings A and C of ponciretin, a flavonoid, and form conventional hydrogen bonds. In contrast, the benzene ring in ring C significantly aids in generating hydrophobic interactions with residues within the active site of BACE1.
2. Regarding chrysophanol and danthron, which are anthraquinone compounds, the hydroxyl groups located within rings A, B, and C, along with the oxygen atom situated within ring B, participate in establishing conventional hydrogen bonds. Furthermore, rings A and C partake in hydrophobic interactions with the residues of the receptor.
3. In the instance of N-p-coumaroyltyramine, categorized as a cinnamic acid, the hydroxyl functional group on the aromatic rings and the oxygen atom within the carboxylic acid display conventional hydrogen bonds. Additionally, the amino (NH) functional group significantly contributes to the creation of stabilizing salt bridges with the residues located within the BACE1 catalytic cleft.

Finally, and without diminishing its significance, it is imperative to validate the inhibitory effects of these compounds through *in vitro* experimentation, followed by subsequent clinical trials.

## 5. Conclusion

The results of this study suggest that 18 organic compounds can penetrate the BBB, with ponciretin, danthron, chrysophanol, and N-p-coumaroyltyramine exhibiting significant binding affinity to the BACE1 active site, and *Ki* values in the nanomolar range. The *ΔG*_binding_ values between these compounds and the BACE1 catalytic domain were estimated to be −8.78, −8.30, −8.21, and −8.51 kcal/mol, respectively. Further examinations revealed that the positioned configurations of these compounds, particularly ponciretin, and N-p-coumaroyltyramine, exhibited consistent stability throughout an MD simulation lasting approximately 50 ns. Although, further *in vivo* research is required to confirm these findings, these compounds may be promising candidates for developing preventive treatments against AD by inhibiting A*β* generation.

## Figures and Tables

**Table 1:** The binding energy and *Ki* values between the BACE1 active site and 18 herbal isolates and eight positive control drugs were estimated in this study.

PubChem ID	Ligand name	Binding energy (kcal/mol)	*Ki*
A. Flavonoids			
25201019	Ponciretin	−8.78	366.56 nM
5281607	Chrysin	−8.01	1.34 *μ*M
629440	Hemileiocarpin	−7.97	1.43 *μ*M
638278	Isoliquiritigenin	−7.66	2.42 *μ*M
124052	Glabridin	−7.55	2.93 *μ*M
5318998	Licochalcone A	−7.04	6.94 *μ*M
10680	Flavone	−6.85	9.49 *μ*M
5280378	Formononetin	−6.55	15.77 *μ*M
B. Anthraquinones			
2950	Danthron	−8.30	824.47 nM
10208	Chrysophanol	−8.21	965.54 nM
6293	Alizarin	−7.64	2.49 *μ*M
124062	Rubiadin	−6.13	31.99 *μ*M
C. Cinnamic acid derivatives			
5372945	N-p-Coumaroyltyramine	−8.51	573.79 nM
5281787	Caffeic acid phenethyl ester	−7.86	1.74 *μ*M
637540	o-Coumaric acid	−6.38	20.89 *μ*M
445858	Ferulic acid	−6.03	37.96 *μ*M
637542	p-Coumaric acid	−5.97	41.75 *μ*M
444539	Cinnamic acid	−5.74	62.34 *μ*M

Drugbank ID	Ligand name	Binding energy (kcal/mol)	*Ki*

D. Control positive compounds			
DB07206	6-[2-(1H-INDOL-6-YL)ETHYL]PYRIDIN-2-AMINE	−10.64	15.91 nM
DB07734	N-(1-benzylpiperidin-4-yl)-4-sulfanylbutanamide	−8.8	354.01 nM
DB07345	4-(2-aminoethyl)-2-cyclohexylphenol	−8.66	452.53 nM
DB07303	N ∼ 3∼−[3-(5-METHOXYPYRIDIN-3-YL)BENZYL]PYRIDINE-2,3-DIAMINE	−8.53	554.33 nM
DB07874	(6S)-2-amino-6-(3′-methoxybiphenyl-3-yl)-3,6-dimethyl-5,6-dihydropyrimidin-4(3H)-one	−7.94	1.52 *μ*M
DB07573	(2S)-1-(2,5-dimethylphenoxy)-3-morpholin-4-ylpropan-2-ol	−7.16	5.68 *μ*M
DB07110	4-(4-FLUOROBENZYL)PIPERIDINE	−7.1	6.28 *μ*M
DB07346	4-(2-aminoethyl)-2-ethylphenol	−6.98	7.60 *μ*M

*Ki*, inhibition constant; BACE1, *β*-site amyloid precursor protein cleaving enzyme 1.

**Table 2:** Details of energies between top-ranked herbal isolates and BACE1 catalytic site achieved from the AutoDock software.

Ligand name	Intermolecular energy (kcal/mol)	Total internal energy (kcal/mol)	Torsional free energy (kcal/mol)	Unbound system's energy (kcal/mol)	Free binding energy (kcal/mol)
Ponciretin	−8.57	−2.26	1.49	−0.55	−8.78
Chrysophanol	−7.54	−2.18	1.19	−0.31	−8.21
Danthron	−7.67	−2.13	1.19	−0.31	−8.30
N-p-Coumaroyltyramine	−11.03	−0.63	2.98	−0.17	−8.51

BACE1, *β*-site amyloid precursor protein cleaving enzyme 1.

**Table 3:** Interaction modes between BACE1 active site and its top-ranked herbal inhibitors.

Ligand name	Hydrogen bond (distance Å)	Hydrophobic interaction (distance Å)	Electrostatic (distance Å)
Ponciretin (before MD)	Asn111 (4.39 classical); Lys107 (4.49 classical)	Ile110 (4.23 alkyl); Lys107 (4.72 alkyl); Phe47 (5.70 pi-alkyl); His45 (7.6 pi-alkyl)	NA
Ponciretin (after MD)	**Asn111** (4.18 classical); Phe109 (3.93 nonclassical, 4.66 nonclassical)	**Lys107** (6.2 alkyl); **Phe47** (6.69 pi-alkyl)	NA
Chrysophanol (before MD)	Asp32 (4.74 classical; 4.75 classical)	Arg128 (6.39 alkyl); Val69 (6.34 alkyl); Tyr198 (6.07)	NA
Chrysophanol (after MD)	Gly34 (4.11 classical)	NA	NA
Danthron (before MD)	Asp106 (3.04 classical, 3.27 classical, 4.73 classical); Lys107 (3.44 classical)	Lys107 (4.63 alkyl); Phe47 (6.6 pi-alkyl, His45 (7.29 pi-alkyl)	NA
Danthron (after MD)	**Asp106** (4.32 classical); Lys107 (4.02 nonclassical)	NA	NA
N-p-Coumaroyltyramine (before MD)	Phe108 (3.65 classical); Tyr231 (3.62 classical); Asp228 (4.5 classical); Asp32 (4.22 salt-bridge)	Leu30 (5.62 alkyl); Tyr71 (7.13 pi-alkyl)	Asp228 (5.59 salt bridge); Asp32 (4.72 salt bridge)
N-p-Coumaroyltyramine (after MD)	**Phe108** (4.86 classical); Asp32 (4.46 nonclassical); Asp228 (4.55 nonclassical)	Trp115 (7.65 pi-alkyl)	**Asp32** (4.38 salt bridge); **Asp228** (4.12 salt bridge)
6-[2-(1H-INDOL-6-YL)ETHYL]PYRIDIN-2-AMINE (Before MD)	Lys107 (4.54 classical); Asp106 (3.45 classical); Ser105 (4.33 classical)	Ile110 (4.31 pi-alkyl, 5.10 pi-alkyl)	NA
6-[2-(1H-INDOL-6-YL)ETHYL]PYRIDIN-2-AMINE (after MD)	**Ser105** (5.00 classical); **Asp106** (3.43 classical)	Lys107 (4.37 pi-alkyl)	Lys107 (4.37 pi-charge)

The bold typeface is employed to exhibit stable interactions before and after MD simulations. BACE1, *β*-site amyloid precursor protein cleaving enzyme 1; MD, molecular dynamics; NA, not available.

## Data Availability

The datasets used and/or analyzed during the current study are available from the corresponding author upon reasonable request.

## Supplementary Materials

Supplementary Materials Table S1: a total of 79 herbal compounds were evaluated for BBB permeability.

## References

[1] Hou Y., Dan X., Babbar M. (2019). Ageing as a risk factor for neurodegenerative disease. *Nature Reviews Neurology*.

[2] Kritsilis M., Rizou S. V., Koutsoudaki P. N., Evangelou K., Gorgoulis V. G., Papadopoulos D. (2018). Ageing, cellular senescence and neurodegenerative disease. *International Journal of Molecular Sciences*.

[3] Ehrenberg A. J., Khatun A., Coomans E. (2020). Relevance of biomarkers across different neurodegenerative diseases. *Alzheimer’s Research & Therapy*.

[4] Hampel H., Vassar R., De Strooper B. (2021). The *β*-secretase BACE1 in Alzheimer’s disease. *Biological Psychiatry*.

[5] Coimbra J. R. M., Marques D. F. F., Baptista S. J. (2018). Highlights in BACE1 inhibitors for Alzheimer’s disease treatment. *Frontiers in Chemistry*.

[6] Lange J., Lunde K. A., Sletten C. (2015). Association of a *BACE1* gene polymorphism with Parkinson’s disease in a Norwegian population. *Parkinson’s Disease*.

[7] Olasehinde T. A., Olaniran A. O., Okoh A. I. (2019). Macroalgae as a valuable source of naturally occurring bioactive compounds for the treatment of Alzheimer’s disease. *Marine Drugs*.

[8] Fakhri S., Pesce M., Patruno A. (2020). Attenuation of Nrf2/Keap1/ARE in Alzheimer’s disease by plant secondary metabolites: a mechanistic review. *Molecules*.

[9] Arruda H. S., Neri-Numa I. A., Kido L. A., Maróstica Júnior M. R., Pastore G. M. (2020). Recent advances and possibilities for the use of plant phenolic compounds to manage ageing-related diseases. *Journal of Functional Foods*.

[10] Garcia-Moreno J. C., Porta de la Riva M., Martínez-Lara E., Siles E., Cañuelo A. (2019). Tyrosol, a simple phenol from EVOO, targets multiple pathogenic mechanisms of neurodegeneration in a C. elegans model of Parkinson’s disease. *Neurobiology of Aging*.

[11] Yener I., Kocakaya S. O., Ertas A. (2020). Selective *in vitro* and *in silico* enzymes inhibitory activities of phenolic acids and flavonoids of food plants: relations with oxidative stress. *Food Chemistry*.

[12] Taherkhani A., Orangi A., Moradkhani S., Khamverdi Z. (2021). Molecular docking analysis of flavonoid compounds with matrix metalloproteinase- 8 for the identification of potential effective inhibitors. *Letters in Drug Design & Discovery*.

[13] Moradkhani S., Farmani A., Saidijam M., Taherkhani A. (2021). COVID-19: docking-based virtual screening and molecular dynamics study to identify potential SARS-CoV-2 spike protein inhibitors from plant-based phenolic compounds. *Acta Virologica*.

[14] Campora M., Francesconi V., Schenone S., Tasso B., Tonelli M. (2021). Journey on naphthoquinone and anthraquinone derivatives: new insights in Alzheimer’s disease. *Pharmaceuticals*.

[15] Jung H. A., Ali M. Y., Jung H. J., Jeong H. O., Chung H. Y., Choi J. S. (2016). Inhibitory activities of major anthraquinones and other constituents from *Cassia obtusifolia* against *β*-secretase and cholinesterases. *Journal of Ethnopharmacology*.

[16] Adisakwattana S. (2017). Cinnamic acid and its derivatives: mechanisms for prevention and management of diabetes and its complications. *Nutrients*.

[17] Anantharaju P. G., Gowda P. C., Vimalambike M. G., Madhunapantula S. R. V. (2016). An overview on the role of dietary phenolics for the treatment of cancers. *Nutrition Journal*.

[18] Wadman M. (2023). FDA no longer needs to require animal tests before human drug trials. *Science*.

[19] Daina A., Michielin O., Zoete V. (2017). SwissADME: a free web tool to evaluate pharmacokinetics, drug-likeness and medicinal chemistry friendliness of small molecules. *Scientific Reports*.

[20] Masumi M., Noormohammadi F., Kianisaba F., Nouri F., Taheri M., Taherkhani A. (2022). Methicillin-resistant *Staphylococcus aureus*: docking-based virtual screening and molecular dynamics simulations to identify potential penicillin-binding protein 2a inhibitors from natural flavonoids. *International Journal of Microbiology*.

[21] Taherkhani A., Ghonji F., Mazaheri A., Lohrasbi M. P., Mohamadi Z., Khamverdi Z. (2021). Identification of potential glucosyltransferase inhibitors from cinnamic acid derivatives using molecular docking analysis: a bioinformatics study. *Avicenna Journal Clinical Microbiology and Infection*.

[22] Shimizu H., Tosaki A., Kaneko K., Hisano T., Sakurai T., Nukina N. (2008). Crystal structure of an active form of BACE1, an enzyme responsible for amyloid *β* protein production. *Molecular and Cellular Biology*.

[23] Rose P. W., Prlić A., Altunkaya A. (2017). The RCSB protein data bank: integrative view of protein, gene and 3D structural information. *Nucleic Acids Research*.

[24] Guex N., Peitsch M. C., Schwede T. (2009). Automated comparative protein structure modeling with SWISS-MODEL and Swiss-PdbViewer: a historical perspective. *ELECTROPHORESIS*.

[25] Hassan M., Shahzadi S., Seo S. Y., Alashwal H., Zaki N., Moustafa A. A. (2018). Molecular docking and dynamic simulation of AZD3293 and solanezumab effects against BACE1 to treat Alzheimer’s disease. *Frontiers in Computational Neuroscience*.

[26] Taherkhani A., Moradkhani S., Orangi A., Jalalvand A., Khamverdi Z. (2021). In silico study of some natural anthraquinones on matrix metalloproteinase inhibition. *Research Journal of Pharmacognosy*.

[27] Wishart D. S., Feunang Y. D., Guo A. C. (2018). DrugBank 5.0: a major update to the DrugBank database for 2018. *Nucleic Acids Research*.

[28] Crascì L., Basile L., Panico A. (2017). Correlating *in vitro* target-oriented screening and docking: inhibition of matrix metalloproteinases activities by flavonoids. *Planta Medica*.

[29] Jayaraj J. M., Reteti E., Kesavan C., Muthusamy K. (2021). Structural insights on Vitamin D receptor and screening of new potent agonist molecules: structure and ligand-based approach. *Journal of Biomolecular Structure and Dynamics*.

[30] Tasleem M., Ishrat R., Islam A., Ahmad F., Hassan M. I. (2014). Structural characterization, homology modeling and docking studies of ARG674 mutation in MyH8 gene associated with trismus-pseudocamptodactyly syndrome. *Letters in Drug Design & Discovery*.

[31] Khan S., Bhardwaj T., Somvanshi P. (2018). Inhibition of C298S mutant of human aldose reductase for antidiabetic applications: evidence from in silico elementary mode analysis of biological network model. *Journal of Cellular Biochemistry*.

[32] Taherkhani A., Moradkhani S., Orangi A., Jalalvand A., Khamverdi Z. (2021). Molecular docking study of flavonoid compounds for possible matrix metalloproteinase-13 inhibition. *Journal of Basic and Clinical Physiology and Pharmacology*.

[33] Jankovic J. (2008). Parkinson’s disease: clinical features and diagnosis. *Journal of Neurology, Neurosurgery & Psychiatry*.

[34] Svenningsson P., Westman E., Ballard C., Aarsland D. (2012). Cognitive impairment in patients with Parkinson’s disease: diagnosis, biomarkers, and treatment. *The Lancet Neurology*.

[35] Mahmud S., Parves M. R., Riza Y. M. (2020). Exploring the potent inhibitors and binding modes of phospholipase A2 through in silico investigation. *Journal of Biomolecular Structure and Dynamics*.

[36] Yusof N. I. S. M., Abdullah Z. L., Othman N., Fauzi F. M. (2022). Structure–activity relationship analysis of flavonoids and its inhibitory activity against BACE1 enzyme toward a better therapy for Alzheimer’s disease. *Frontiers in Chemistry*.

[37] Lin L., Luo L., Zhong M. (2019). Gut microbiota: a new angle for traditional herbal medicine research. *RSC Advances*.

[38] Kang G.-D., Kim D.-H. (2016). Poncirin and its metabolite ponciretin attenuate colitis in mice by inhibiting LPS binding on TLR4 of macrophages and correcting Th17/Treg imbalance. *Journal of Ethnopharmacology*.

[39] Tran T.-S., Tran T.-D., Tran T.-H. (2020). Synthesis, in silico and in vitro evaluation of some flavone derivatives for acetylcholinesterase and BACE-1 inhibitory activity. *Molecules*.

[40] Cho N., Choi J. H., Yang H. (2012). Neuroprotective and anti-inflammatory effects of flavonoids isolated from *Rhus verniciflua* in neuronal HT22 and microglial BV2 cell lines. *Food and Chemical Toxicology*.

[41] Zhang S., Qi Y., Xu Y. (2013). Protective effect of flavonoid-rich extract from *Rosa laevigata* Michx on cerebral ischemia–reperfusion injury through suppression of apoptosis and inflammation. *Neurochemistry International*.

[42] Mohebali N., Shahzadeh Fazeli S. A., Ghafoori H. (2018). Effect of flavonoids rich extract of *Capparis spinosa* on inflammatory involved genes in amyloid-beta peptide injected rat model of Alzheimer’s disease. *Nutritional Neuroscience*.

[43] Shimmyo Y., Kihara T., Akaike A., Niidome T., Sugimoto H. (2008). Flavonols and flavones as BACE-1 inhibitors: structure–activity relationship in cell-free, cell-based and in silico studies reveal novel pharmacophore features. *Biochimica et Biophysica Acta (BBA) - General Subjects*.

[44] Chiou S.-M., Chiu C.-H., Yang S.-T. (2012). Danthron triggers ROS and mitochondria-mediated apoptotic death in C6 rat glioma cells through caspase cascades, apoptosis-inducing factor and endonuclease G multiple signaling. *Neurochemical Research*.

[45] Kwon Y.-S., Koh J.-Y., Song D.-K. (2004). Danthron inhibits the neurotoxicity induced by various compounds causing oxidative damages including *β*-Amyloid (25–35) in primary cortical cultures. *Biological and Pharmaceutical Bulletin*.

[46] Zeng P., Shi Y., Wang X.-M. (2019). Emodin rescued hyperhomocysteinemia-induced dementia and Alzheimer’s disease-like features in rats. *International Journal of Neuropsychopharmacology*.

[47] Li X., Cheng Y., Qin Y. (2022). Chrysophanol exerts neuroprotective effects via interfering with endoplasmic reticulum stress apoptotic pathways in cell and animal models of Alzheimer’s disease. *Journal of Pharmacy and Pharmacology*.

[48] Ghafary S., Nadri H., Mahdavi M. (2020). Anticholinesterase activity of cinnamic acids derivatives: in vitro, in vivo biological evaluation, and docking study. *Letters in Drug Design & Discovery*.

[49] Takahashi T., Miyazawa M. (2011). Serotonin derivatives as inhibitors of *β*-secretase (BACE 1). *Die Pharmazie*.

[50] Takao K., Toda K., Saito T., Sugita Y. (2017). Synthesis of amide and ester derivatives of cinnamic acid and its analogs: evaluation of their free radical scavenging and monoamine oxidase and cholinesterase inhibitory activities. *Chemical and Pharmaceutical Bulletin*.

[51] Lan J.-S., Hou J.-W., Liu Y. (2017). Design, synthesis and evaluation of novel cinnamic acid derivatives bearing *N*-benzyl pyridinium moiety as multifunctional cholinesterase inhibitors for Alzheimer’s disease. *Journal of Enzyme Inhibition and Medicinal Chemistry*.

