# Supplementary material for: BACE1 Inhibition Utilizing Organic Compounds Holds Promise as a Potential Treatment for Alzheimer's and Parkinson's Diseases
Source: Oxid Med Cell Longev. 2024 Feb 22;2024:6654606. doi: 10.1155/2024/6654606 (PMC10904208; doi:10.1155/2024/6654606)
Supplement: Supplementary Materials — Table S1: a total of 79 herbal compounds were evaluated for BBB permeability. [file 6654606.f1.docx]

**Supplementary Table 1.** A total of 79 herbal compounds were evaluated for BBB permeability.

| **A, Flavonoids** | | |
| --- | --- | --- |
| **PubChem ID** | **Ligand name** | **BBB Permeant** |
| 44258853 | Kaempferol 3-rutinoside-7-sophoroside | No |
| 5280805 | Rutin | No |
| 5281600 | Amentoflavone | No |
| 5280343 | Quercetin | No |
| 44258844 | Kaempferol 3-rutinoside-4'-glucoside | No |
| 5280704 | Apigenin-7-glucoside | No |
| 5280804 | Isoquercitrin | No |
| 5281675 | Orientin | No |
| 5318767 | Nicotiflorin | No |
| 442664 | Vicenin-2 | No |
| 72936 | Sophoraflavanone G | No |
| 9911508 | Astragarin | No |
| 5318998 | Licochalcone A | Yes |
| 5353915 | Quercetin-3-rhamnoside | No |
| 5280445 | Luteolin | No |
| 5280637 | Cynaroside | No |
| 5280441 | Vitexin | No |
| 5281672 | Myricetin | No |
| 5280459 | Quercitrin | No |
| 14309735 | Xanthogalenol | No |
| 639665 | XanthohuMol | No |
| 638278 | Isoliquiritigenin | Yes |
| 5281612 | Diosmetin | No |
| 1203 | Epicatechin | No |
| 5317435 | Fustin | No |
| 5280443 | Apigenin | No |
| 10095180 | Kaempferol 7-O-glucoside | No |
| 5281607 | Chrysin | Yes |
| 5281654 | Isorhamnetin | No |
| 5280681 | 3-O-Methylquercetin | No |
| 471 | Dihydroquercetin | No |
| 443639 | Epiafzelechin | No |
| 124052 | Glabridin | Yes |
| 5316673 | Afzelin | No |
| 72281 | Hesperetin | No |
| 5280544 | Herbacetin | No |
| 5282102 | Astragalin | No |
| 629440 | Hemileiocarpin | Yes |
| 5281670 | Morin | No |
| 25201019 | Ponciretin | Yes |
| 5280863 | Kaempferol | No |
| 9064 | Catechin | No |
| 10680 | Flavone | Yes |
| 5281614 | Fisetin | No |
| 439533 | Taxifolin | No |
| 5280378 | Formononetin | Yes |
| **B, Antraquinones** | | |
| **PubChem ID** | **Ligand name** | **BBB Permeant** |
| 442731 | Pulmatin (Chrysophanol 8-O-glucoside) | No |
| 92826 | Sennidin A | No |
| 99649 | Emodin-8-glucoside | No |
| 3220 | Emodin | No |
| 101286218 | Rhodoptilometrin | No |
| 10208 | Chrysophanol | Yes |
| 442753 | Knipholone | No |
| 10459879 | Sennidin B | No |
| 126456371 | Aloe Emodin 8-Glucoside | No |
| 10207 | Aloe-emodin | No |
| 3083575 | Obtusifolin | No |
| 361510 | Emodic acid | No |
| 10168 | Rhein | No |
| 2950 | Danthron | Yes |
| 3663 | Hypericin | No |
| 6683 | Purpurin | No |
| 10639 | Physcion | No |
| 6293 | Alizarin | Yes |
| 2948 | Damnacanthal | No |
| 124062 | Rubiadin | Yes |
| 160712 | Nordamnacanthal | No |
| **C, Cinamic acid derivatives** | | |
| **PubChem ID** | **Ligand name** | **BBB Permeant** |
| 1794427 | Chlorogenic acid | No |
| 5281759 | Caffeic acid 3-glucoside | No |
| 5281792 | Rosmarinic acid | No |
| 5372945 | N-p-Coumaroyltyramine | Yes |
| 5281787 | Caffeic acid phenethyl ester | Yes |
| 6124212 | Cynarin | No |
| 637540 | o-Coumaric acid | Yes |
| 689043 | Caffeic acid | No |
| 445858 | Ferulic acid | Yes |
| 637542 | p-Coumaric acid | Yes |
| 444539 | Cinnamic acid | Yes |
| 637775 | Sinapinic acid | No |

BBB, blood-brain barrier.
